# Increased Abscess Formation and Defective Chemokine Regulation in CREB Transgenic Mice

**DOI:** 10.1371/journal.pone.0055866

**Published:** 2013-02-06

**Authors:** Andy Y. Wen, Elliot M. Landaw, Rachel Ochoa, Michelle Cho, Alex Chao, Gregory Lawson, Kathleen M. Sakamoto

**Affiliations:** 1 Division of Critical Care, Department of Pediatrics, David Geffen School of Medicine at University of California Los Angeles, Los Angeles, California, United States of America; 2 Department of Biomathematics, David Geffen School of Medicine at University of California Los Angeles, Los Angeles, California, United States of America; 3 Division of Pediatric Hematology/Oncology, Lucile Packard Children’s Hospital, Stanford University School of Medicine, Stanford, California, United States of America; 4 Department of Molecular, Cell, and Developmental Biology, University of California Los Angeles, Los Angeles, California, United States of America; 5 Division of Laboratory Animal Medicine, David Geffen School of Medicine at University of California Los Angeles, Los Angeles, California, United States of America; University of California, Riverside, United States of America

## Abstract

Cyclic AMP-response element-binding protein (CREB) is a transcription factor implicated in growth factor-dependent cell proliferation and survival, glucose homeostasis, spermatogenesis, circadian rhythms, and synaptic plasticity associated with memory. To study the phenotype of CREB overexpression *in vivo*, we generated CREB transgenic (TG) mice in which a myeloid specific hMRP8 promoter drives CREB expression. CREB TG mice developed spontaneous skin abscesses more frequently than wild type (WT) mice. To understand the role of CREB in myeloid function and innate immunity, chemokine expression in bone marrow derived macrophages (BMDMs) from CREB TG mice were compared with BMDMs from WT mice. Our results demonstrated decreased Keratinocyte-derived cytokine (KC) in CREB TG BMDMs but not TNFα protein production in response to lipid A (LPA). In addition, mRNA expression of KC and IL-1β (Interleukin)-1β was decreased in CREB TG BMDMs; however, there was no difference in the mRNA expression of TNFα, MCP-1, IL-6 and IL-12p40. The mRNA expression of IL-1RA and IL-10 was decreased in response to LPA. Nuclear factor kappa B (NFκB) expression and a subset of its target genes were upregulated in CREB TG mouse BMDMs. Although neutrophil migration was the same in both CREB TG and WT mice, Nicotinamide adenine dinucleotide phosphate (NADPH) oxidase activity was significantly increased in neutrophils from CREB TG mice. Taken together, CREB overexpression in myeloid cells results in increased abscess formation *in vivo* and aberrant cytokine and chemokine response, and neutrophil function *in vitro*.

## Introduction

CREB is a transcription factor that controls diverse cellular functions, including proliferation, differentiation, and survival [1], [2], [3]. In hematopoietic cells, CREB regulates normal and leukemic cell proliferation and differentiation. Recent evidence suggests that CREB plays a role in immune function by interacting with NFκB activation, inducing macrophage survival, and regulating proliferation and survival of T and B lymphocytes [4]. In previous studies, we demonstrated that transgenic (TG) mice overexpressing CREB in myeloid cells develop myeloproliferative disease after one year of age [5]. In addition, these mice had a higher incidence of skin abscesses. Very little is known about the role of CREB in inflammation and innate immune response.

Suppuration and abscesses of the preputial glands in mice typically occur as long-term complications of fight wounds around the external genitalia [6]. The cause of preputial gland abscesses are infrequently investigated, and little information exists on the bacterial pathogens involved in this type of lesion [6], [7].

NFκB is an important modulator of immunoregulatory gene expression. Prototypical activators of NFκB are Toll Like Receptors (TLRs), which initiate a signaling cascade resulting in NFκB pathway activation and triggering of other important signaling cascades through IFN regulatory factors and MAPKs, including ERK1/2, JNKs, and p38 isoforms [8], [9]. The NFκB pathway can also be activated by proinflammatory cytokines, such as IL-1β, and TNF-α [9], [10]. CREB is known to regulate a number of immune-regulatory genes such as IL-2, IL-6, IL-10, TNF-alpha, cyclooxygenase-2, and macrophage migration-inhibitory factor.

In this paper, we analyzed the relationship between CREB overexpression and its effects on chemokine production in bone marrow macrophages (BMMs) from CREB TG and WT mice. We characterized the frequency and histology of skin abscesses and chemokines that could be responsible for mediating the abnormal inflammatory response in CREB transgenic mice. NFκB expression and target genes in macrophages were also examined. Finally, neutrophil function was investigated to determine the response of myeloid cells that overexpress CREB. Our results demonstrate that CREB overexpression leads to abnormal chemokine production in macrophages and aberrant NADPH oxidase activity in neutrophils.

## Materials and Methods

### CREB Transgenic Mice

Transgenic mice were created in which CREB was overexpressed in Gr-1/Mac-1+ monocytes/macrophages under the control of the hMRP8 promoter. WT and CREB transgenic mice were bred on a C57BL/6 background. Female C57BL/6 mice were used from a breeding colony and additional wild-type C57BL/6 female mice were purchased from BD Bioscience. All mice were maintained and bred at the University of California, Los Angeles, Department of Laboratory Animal Medicine mouse facility (Los Angeles, CA) under specific pathogen-free conditions with an approved institutional animal protocol. This study was carried out in strict accordance with the recommendations in the Guide for the Care and Use of Laboratory Animals of the National Institutes of Health. The protocol was approved by the Committee on Ethics of Animal Experiments of the University of California, Los Angeles (ARC# 1999-069-33).

### Characterization of Infections and Infection Rates

Breeding colony data were compiled from a total of 1832 mice followed from birth until removal from the colony. WT (887) and 945 homozygous CREB transgenic C57BL/6 mice were examined for the development of spontaneous infections, and data were prospectively collected to quantify the number and time of first appearance of each type of infection and to analyze the distribution of affected mice based on presence or absence of CREB transgene, gender, and age.

### Necropsy, Histopathology, and Pathogen Isolation

Mice were euthanized by CO2 asphyxiation followed by cervical dislocation, and complete necropsies were performed as per the approved institutional animal protocol. Infected skin samples and preputial gland abscesses were obtained from sites of infection, then fixed in 10% neutral buffered formalin, embedded in paraffin, sectioned, and stained with hematoxylin and eosin for microscopic examination of inflammation. Bacterial and fungal cultures from swabs and infected-tissues were performed.

### Bone Marrow-derived Macrophage (BMDM) Extraction

Murine BMDMs were obtained by flushing bone marrow cells from femurs and tibias of uninfected 6 to 8 week-old mice. Cells were then cultured for 7 days in DMEM containing 10% FBS, penicillin (100 U/ml), streptomycin (100 µg/ml), and 20% conditioned medium from L929 cells overexpressing macrophage colony-stimulating factor (M-CSF). Following 7 days of growth, the media was changed to DMEM supplemented with 5% HI-FBS, penicillin (100 U/ml), and streptomycin (100 µg/ml) to rest cells for 24 hours prior to stimulation. Bone marrow-derived macrophages in PBS, 0.2% BSA were identified using F4/80 (BD Biosciences, San Jose, CA) and CD11b cell surface markers. Cells were >93% pure, as determined by FACS analysis.

### *In Vitro* Stimulation of Bone Marrow-derived Macrophages

Cultures of bone marrow-derived macrophages were activated by the following TLR agonists: 10 ng/ml lipid A [LPA] (Alexis Biochemicals, San Diego, CA); 0.1 µg/ml palmitoyl-3-cysteine [Pam3CSK4] (Alexis Biochemical, San Diego, CA), and 1000 nM CpG oligodeoxynucleotides [ODN] (InvivoGen, San Diego, CA). Following titration of TLR ligands, bone marrow-derived macrophages were stimulated at 15, 30, 60, 120 minutes, and 24 hours after stimulation, then assayed for production and expression of TNF-α and KC.

### Enzyme-linked Immunosorbent Assays (ELISA)

KC and TNF-α were measured by commercially available ELISA kits (OptEIA kit, BD Biosciences, San Jose, CA) and according to manufacturer’s instructions. Outcomes were assessed as optical densities using a V_max_ kinetic plate reader (Molecular Devices, Menlo Park, CA).

### Quantitative Detection of Murine Chemokine and Cytokine mRNA and NFkB Target Genes

Total mRNA was isolated from mouse bone marrow macrophages using TRIzol (Invitrogen, Grand Island, NY), and cDNA was synthesized using the SuperScript III First Strand cDNA Synthesis Kit (Invitrogen, Carlsbad, CA). Quantitative real-time PCR (qRT-PCR) were performed using SYBR Green qRT-PCR reagent (Bio-Rad Laboratories, Hercules, CA) and the following 10 uM sense and antisense primers, respectively: murine KC 5′-CACTGCACCCAAACCGAAGT-3′ (forward) and 5′-GGACAATTTTCTGAACCAAGGG-3′ (reverse); IκBα 5′-CTGCAGGCCACCAACTACAA-3′ (forward) and 5′-CAGCACCCAAAGTCACCAAGT-3′ (reverse); TNF-α 5′-GGTGCCTATGTCTCAGCCTCTT-3′ (forward) and 5′-CGATCACCCCGAAGTTCGTA-3′ (reverse); and MIP-2 5′-AGCTACATCCCACCCACACAG-3′ (forward) and 5′-AAAGCCATCCGACTGCATCT-3′ (reverse).

Sequences of primers for NFkB target genes (all primers are in the 5′–>3′ direction):

CCL2 F: TAAAAACCTGGATCGGAACCAAA.

CCL2 R: GCATTAGCTTCAGATTTACGGGT.

CCL5 F: GCTGCTTTGCCTACCTCTCC.

CCL5 R: TCGAGTGACAAACACGACTGC.

CSF1 F: GTGTCAGAACACTGTAGCCAC.

CSF1 R: TCAAAGGCAATCTGGCATGAAG.

Irf1 F: ATGCCAATCACTCGAATGCG.

Irf1 R: CCTGCTTTGTATCGGCCTGT.

NFκB F: ATGGCAGACGATGATCCCTAC.

NFκB R: CGGAATCGAAATCCCCTCTGTT.

All experiments were performed in triplicate, analyzed by the standard curve method, and standardized to the housekeeping gene glyceraldehyde-3-phosphate dehydrogenase (GAPDH) on an ABI Prism 7700 sequence detection system (Applied Biosystems, Foster City, CA) according to manufacturer’s instructions.

### Western Blot Analysis

BMDMs, grown on 60 mm dishes or filters, were rinsed in cold PBS and placed on ice in 400 µl lysis buffer per well (1% Triton X-100, 150 mM NaCl, 20 mM Tris-HCl, pH 7.5, 2 mM EDTA, containing 10 µg/ml leupeptin, 10 µg/ml aprotinin, 1 mM sodium fluoride, 1 mM sodium orthovanadate, and 2 mM PMSF). Lysates were centrifuged (12,000×g, 15 min at 4°C), and protein concentration in each supernatant was determined by colorimetric Bradford protein assay (Bio-Rad, Hercules, CA). Proteins (per lane, 15–25 µg) from the resulting supernatants were heated (85°C, 2 min), Boiling SDS-Laemmli method was used for all Western blot analyses. Protein lysates were separated on a 10% SDS-polyacrylamide gel. Immunoblot was performed with anti-CREB (UBI, New York, NY), or anti-actin antisera (Santa Cruz Biotechnology, Santa Cruz, CA) as described previously. All experiments were performed three times [5]. To quantify the bands obtained by Western blot analysis, we applied ImageJ software based analysis (http://rsb.info.nih.go/ij/). The area under the curve (AUC) of the specific signal was corrected for he AUC of the loading control. The value for the ‘Solv’ condition was set as 1 and other conditions were recalculated correspondingly to allow ratio comparisons.

### Statistical Analysis

Parametric data were summarized as mean ± SEM and analyzed using unpaired Student’s *t* test. For mouse colony data, the time to first appearance of an abscess or non-abscess skin infection/dermatitis was assessed by Kaplan-Meier methods to take into account censoring and the variation in follow-up times among mice in the breeding colonies. Comparisons between groups were made by a log-rank test. Follow-up for each mouse was from birth until the date of first appearance of an abscess or skin infection while in the colony, or until the last date it was still in the colony healthy and infection-free. Removal of an infection-free mouse from the colony (e.g. for use in a different study or because of illness without infection) terminated follow-up and was considered a censoring event. We assumed that such censoring was independent of the risk for developing abscesses. All analyses were conducted using GraphPad Prism Version 5.0a. Unless stated otherwise, *p*<0.05 was considered statistically significant for all data.

### Nitro Blue Tetrazolium (NBT) Assays

Neutrophils from CREB or WT bone marrow were incubated with 10 µM formyl-Leu-Met-Phe (fMLP) and 0.125% NBT for 25 minutes at 37°C. The cells were spun onto cytospins and countered stained with 0.5% Safranin-O (Sigma, Inc.) in 20% ethanol for 5 minutes at room temperature. WT = wild-type mice, CREB = CREB transgenic mice. P-values are represented above the data bars, * p<0.05, ** p<0.01. Error bars represent the standard error.

## Results

### CREB Transgenic Mice have an Increased Incidence of Abscess Formation and Dermatitis

We observed that CREB TG mice developed skin abscesses more frequently than WT controls. Affected mice were photographed and histologic examinations were performed. The tissue histology demonstrated that mice with preputial gland abscesses contained a suppurative infiltrate within the lumen of the preputial gland (Figure S1A). These sites of infection displayed hyperplasia of the squamous epithelial cell lining, keratin plugs, and areas of surrounding edema (Figure S1B). Neutrophils were observed in various stages of transmigration from capillaries through the vascular epithelium to the sites of infection (Figure S1B). Lymphocytes and plasma cells were noted adjacent to sebaceous gland ducts suggestive of acute or chronic inflammation. Other mice with ulcerative dermatitis on visual examination showed clear histologic evidence of disruption of the dermal and epidermal layers in areas adjacent to the mouse genitalia (data not shown).

**Figure 1:**
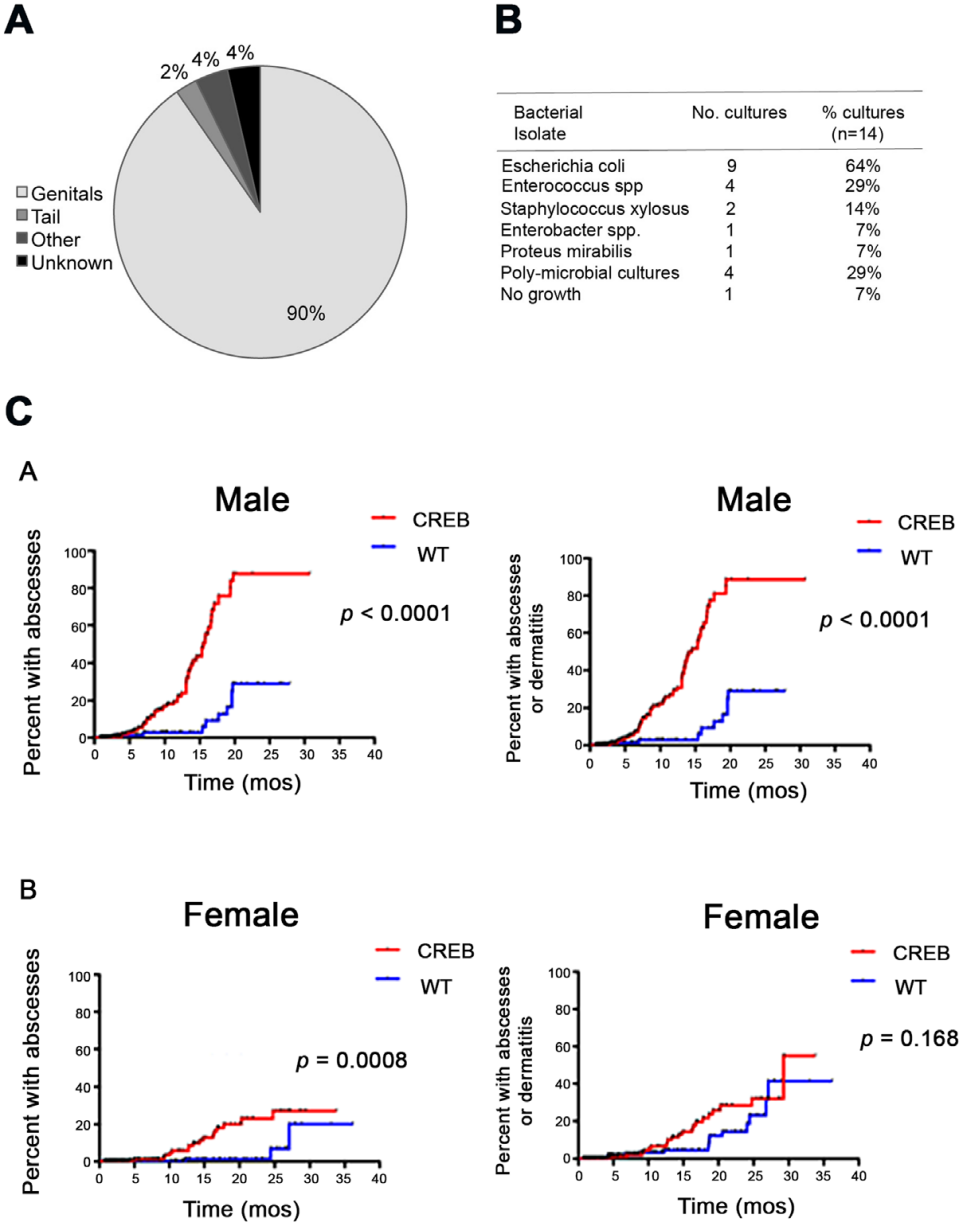
Comparison of abscess location and types of infection. (A) Numbers of mice that developed skin and soft-tissue abscesses were evaluated and the location of abscesses was recorded. The majority (90%) of skin and soft-tissue abscesses were located in the genital region. The remaining abscesses were discovered on the tail (2%), involved the eyes, kidney, and abdomen (4%), or not recorded (4%). (B) Types of bacteria identified in 14 abscesses cultured from CREB TG mice, the numbers of positive cultures, and percent of each type of bacteria are listed. (C) Estimated Cumulative Incidence of skin infections in CREB TG and WT mice. Kaplan-Meier analysis was performed to take censoring into account in estimating the cumulative risk with age of developing a first skin abscess or non-abscess dermatitis in CREB TG and WT, if male and female mice could be followed indefinitely under breeding colony conditions. (A) CREB TG males developed skin abscesses alone earlier and with a higher overall risk than WT male mice (p<0.0001 by log-rank test). Similarly, CREB TG male mice were at greater risk of developing abscesses or non-abscess dermatitis than WT mice (p<0.0001). (B) CREB TG female mice developed skin abscesses earlier than WT female mice (p = 0.0008). However, the time to first appearance of abscess or non-abscess dermatitis did not appear significantly different between CREB TG female and WT female mice (p = 0.168).

To characterize the incidence and frequency of skin abscesses in CREB TG mice compared to WT mice, we followed 887 C57BL/6 WT and 945 homozygous CREB TG mice while they remained in a controlled breeding colony environment (Table 1). The observed net frequency of skin abscess was greater in CREB TG mice than in WT mice in both female mice (4.1% vs. 0.47%) and males (10.4% vs. 4.0%). The majority of abscesses occurred in the mouse genital regions (Figure 1A). Among the bacteria identified in 14 abscesses that were cultured, Escherichia coli was the most prevalent at 64% (Figure 1B). Only one abscess showed no bacterial growth (Figure 1B).

**Table 1:** Characteristics of mice and analysis of abscess formation and dermatitis.

Characteristic	MALES		FEMALES	
	CREB TG	C57BL/6 (WT)	CREB TG	C57BL/6 (WT)
No. abscesses	55	10	17	3
No. skin infections/dermatitis	10	2	6	13
n	529	248	416	639
% abscesses*	10.4%	4.0%	4.1%	0.47%
	p<0.0001		p = 0.0008	
% abscesses & dermatitis*	12.3%	4.8%	5.5%	2.5%
	p<0.0001		p = 0.17	
Median follow-up (months)	3.4	3.2	3.5	3.0
75% follow-up (months)	6.8	9.4	8.4	6.1
90% follow-up (months)	11.9	17.8	17.0	13.7

* raw percentage without regard to length of follow-up; p-value comparing TG to WT determined by Kaplan-Meier analysis with log-rank test.

The majority of animals were removed from the colony while still healthy (e.g., for use in other experimental studies), resulting in median follow-up times less than 4 months (Table 1). To control for the great variation in follow-up times and high percentage of censored observations, we used Kaplan-Meier methods to estimate what would be the cumulative risk of first abscess formation as a function of age if all mice could be followed within the colony (Figure 1C, left panels). These analyses showed that CREB TG mice are expected to develop skin abscesses earlier and more frequently than WT mice in both female (p = 0.0008 by log-rank test) and male (p<0.0001) populations. When the definition of skin infection was extended to include abscess or non-abscess infection/dermatitis (Figure 1C, right panels), CREB TG male mice continued to show earlier and more frequent infection than WT male (p<0.0001), but the distinction in female mice between CREB TG and WT was not significant (p = 0.17).

### Expression of CREB, CXC Chemokine KC, and MCP-1 Following TLR Stimulation

**Figure 2:**
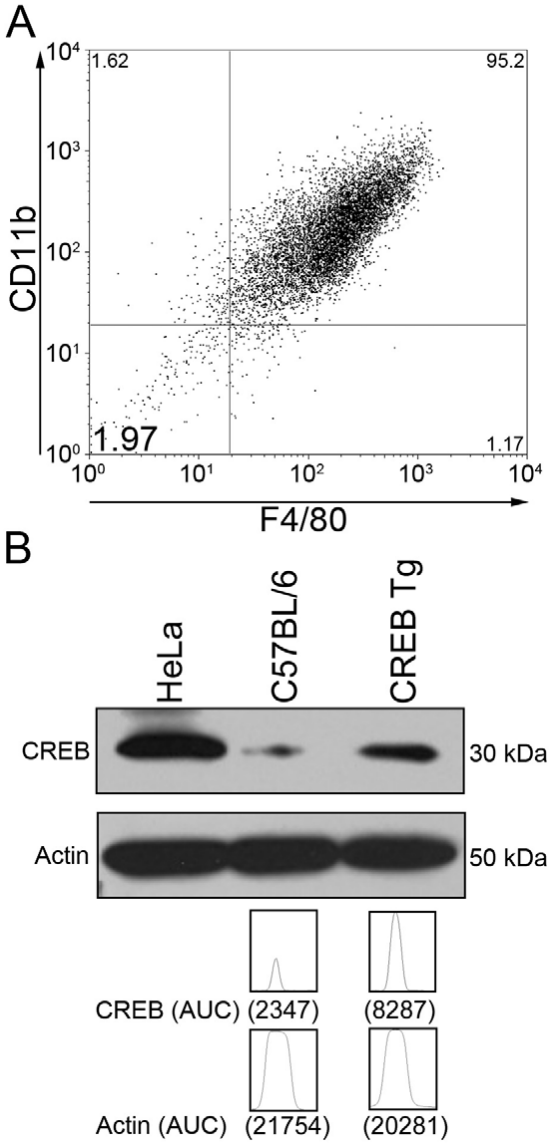
Purification of BMDMs and expression of CREB. (A) Whole bone marrow was obtained from CREB TG and WT mice. Flow cytometry was performed after staining cells for CD11b and F4/80 cell surface markers confirming 93% CD11b+/F4/80+ BMDM population. (B) Western blot analysis was performed with CREB antisera (UBI, Upstate, NY) and normalization for Actin expression (Santa Cruz Biotechnology, Santa Cruz, CA). To quantitate the band, ImageJ software was used (http://rsb.info.nih.go/ij/). CREB TG mice BMDMs had 4-fold greater CREB expression than WT mice.

To study the expression of chemokines in BMDMs from CREB TG or WT mice, we extracted whole bone marrow and cultured cells in L929 conditioned media for 7 days followed by purification by sorting for CD11b+/F4/80+ cells (Figure 2A). To verify CREB overexpression, we performed Western blot analysis. CREB expression was 4-fold higher in BMDMs from CREB TG compared to WT mouse (Figure 2B).

**Figure 3:**
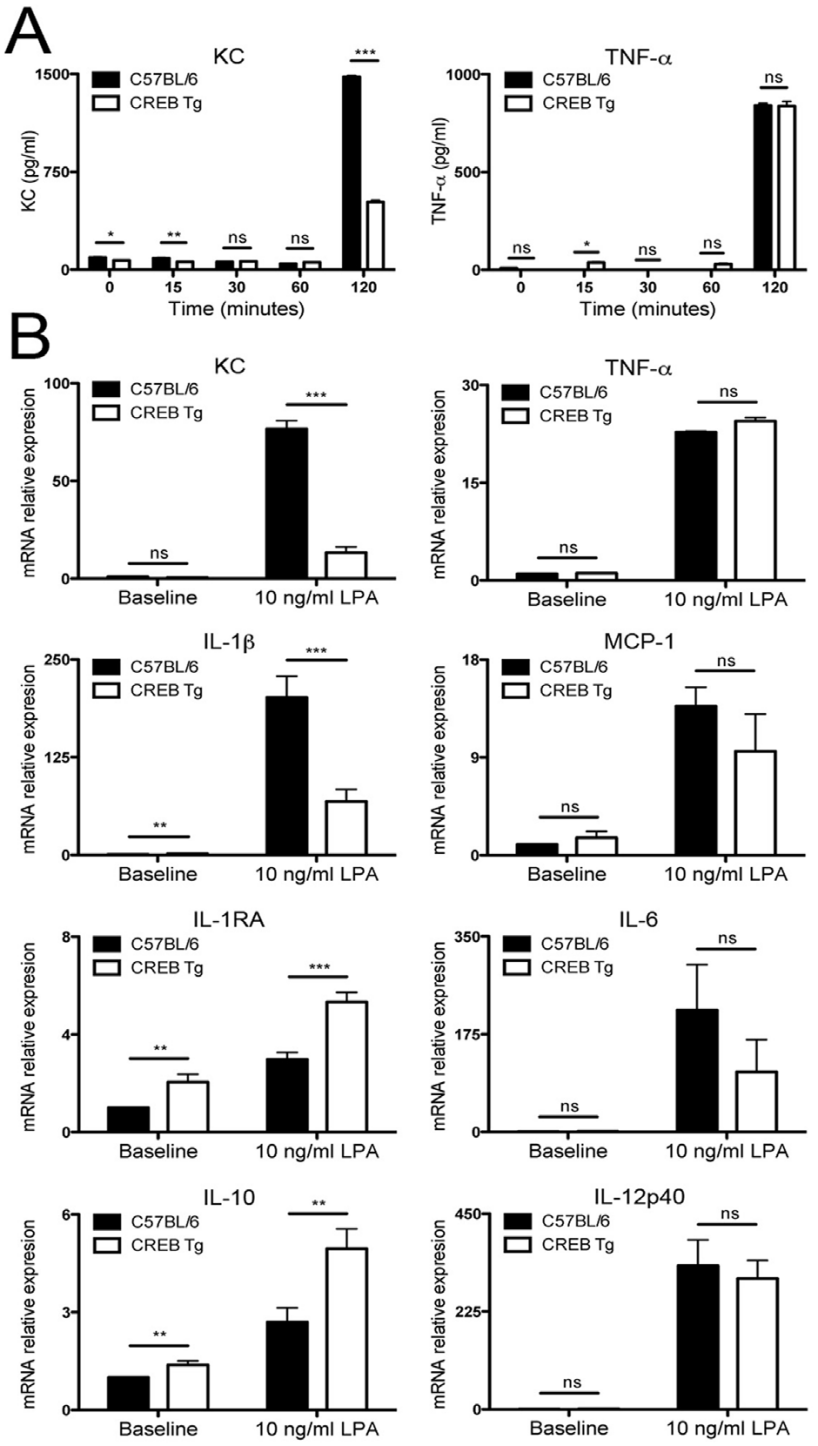
CREB overexpression results in reduced LPA-induced KC production and differential chemokine and cytokine expression. (A) ELISA were performed to quantify KC and TNF-α production and (B) qRT-PCR was performed to compare mRNA expression in BMDMs isolated from CREB TG or WT mice exposed for 2 hours to 10 ng/ml LPA. KC and TNF-α production was measured using ELISA and mRNA levels were measured using qRT-PCR and normalized to GAPDH mRNA levels. The data represent an average ± SEM of four mice analyzed in triplicate for ELISA and three independent experiments with three mice each for qRT-PCR experiments; *p<0.05, **p<0.01, ***p<0.001, ns means “not significant”.

The KC promoter contains a proximal cAMP Response Element (CRE) site that is highly conserved, and identical CRE sequences are present in other CXC chemokine promoters [11]. *In vitro* analysis was performed to characterize the effect of CREB overexpression on production of CXC chemokine KC by BMDMs. Results of chemokine ELISA analysis of WT and CREB transgenic mice with and without LPA stimulation are shown in Figure 3A. Following stimulation by LPA, KC production reached maximal levels by 2 hours where a 3-fold decrease of KC was detected in CREB transgenic BMDMs when compared with WT mice (Figure 3A; n = 4; *p*<0.001). Analysis of KC mRNA expression in BMDMs from CREB TG and WT mice showed a 5-fold decrease in KC expression in CREB transgenic mouse BMDMs stimulated with LPA compared with BMDMs extracted from WT mice (Figure 3B; *p*<0.001). MCP-1 is a CC chemokine that induces monocyte, NK cell, and dendritic cell migration. Comparison of CREB TG and WT mouse BMDMs showed that LPA signaling through TLR4 did not account for a significant change in mRNA expression of MCP-1 (Figure 3B, n = 9; *p*>0.05). In conclusion, early expression of KC, but not MCP-1, was inhibited by CREB overexpression in bone marrow-derived macrophages.

### Effects of CREB Overexpression on Pro- and Anti-inflammatory Cytokines after TLR Stimulation

We next separately investigated various chemokine and cytokine responses to LPA stimulation in BMDMs to discern whether CREB overexpression disrupts immune signaling. Stimulation of CREB TG and WT BMDMs with LPA showed that CREB overexpression resulted in a 3-fold decrease in pro-inflammatory cytokine IL-1β expression and a statistically significant increases in expression of anti-inflammatory cytokines IL-1RA and IL-10 (Figure 3B; n = 9; *p*<0.001). Analysis of TNF-α, IL-6, and IL-12p40 expression showed no significant difference in TNF-α levels (Figure 3A; n = 4; *p*>0.05). Although reduced IL-6 and IL-12p40 expression were observed in CREB TG mouse BMDMs, this was not statistically significant (Figure 3B; n = 9; *p*>0.05). In summary, our results indicate that CREB TG mouse BMDMs have decreased IL-1β and increased IL1-RA and IL-10 expression.

**Figure 4:**
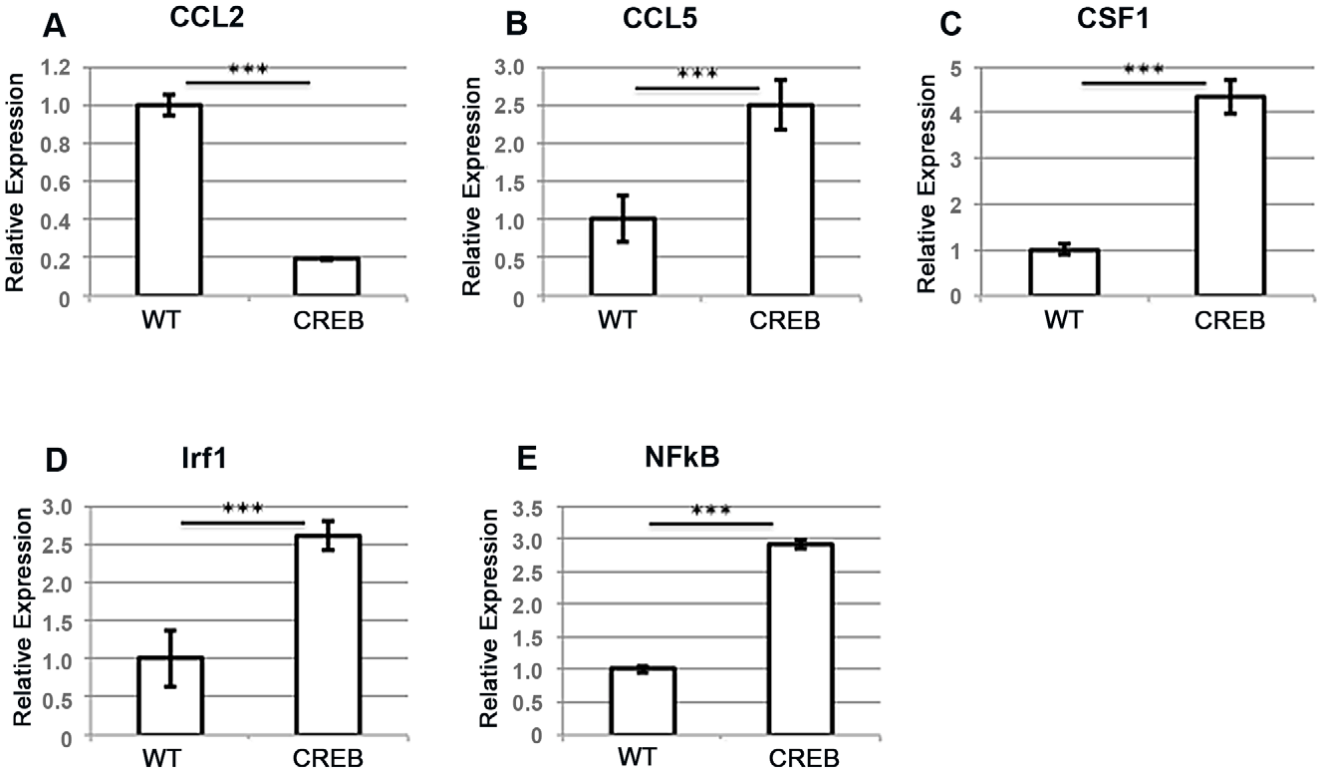
Expression of known nuclear factor kappa B (NFκB) target genes. (A-E) qRT-PCR was performed with RNA from BMDMs from CREB and WT mice. Chemokine ligand 5 (CCL5), Colony Stimulating Factor 1 or Macrophage Colony Stimulating Factor (CSF1 or M-CSF), Interferon Factor 1 (IRF1), and NFkB were upregulated in CREB BMDMs (***p<0.005). Chemokine ligand 2 (CCL2) expression was downregulated. The data represent an average ± SEM of four mice analyzed in triplicate for ELISA and two independent experiments. ***p<0.001.

To further investigate the potential mechanism of increased absence and inflammation in CREB TG mice, we examined the expression of NFκB and known NFκB target genes in BMDMs from CREB TG and WT mice. Previous studies reported that CREB may have an inhibitory effect on NFκB activity. The website for these genes was published on http://www.sabiosciences.com/chipqpcr product/HTML/GM-025A.html. Several NFκB target genes were demonstrated to be upregulated in BMDMs from CREB TG compared WT mice (Figure 4). Chemokine ligand 5 (CCL5), Colony Stimulating Factor 1 or Macrophage Colony Stimulating Factor (CSF1 or M-CSF), Interferon Factor 1 (IRF1), and NFkB were upregulated in CREB BMDMs and found to be statistically significant (p<0.005). However, Chemokine ligand 2 (CCL2) expression was downregulated, suggesting that only a subset of NFκB target genes were upregulated in BMDMs from CREB TG mice. All of these genes have been implicated in macrophage chemotaxis, function, or differentiation.

### Effects of CREB Overexpression on Myeloid Cell Function

**Figure 5:**
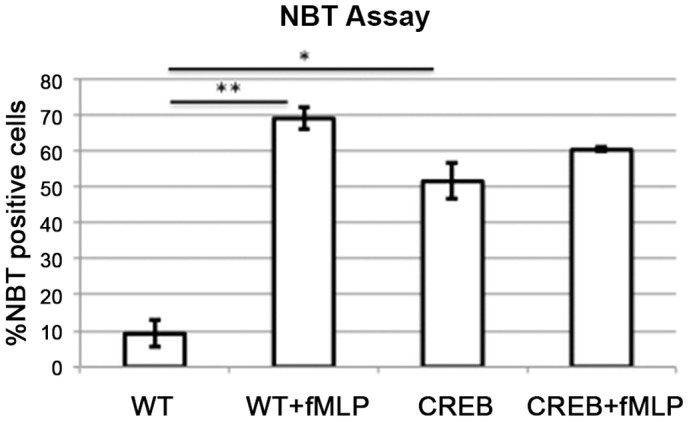
NBT Oxidase Activity. Neutrophils from CREB TG or WT bone marrow were incubated with 10 uM formyl-Leu-Met-Phe (fMLP) and 0.125% NBT. WT = wild type mice, CREB = CREB TG mice. P-values are represented above the data bars, * p<0.05, ** p<0.01. Error bars represent the standard error.

To examine the effects of CREB overexpression on neutrophil function, we performed nitro blue tetrazolium (NBT) assays with neutrophils isolated from CREB TG or WT mice that did not have abscesses and were not infected (Figure 5). The neutrophils had increased basal NBT activity almost equal to cells treated with fMLP, but the difference of 10% was not statistically significant (p = 0.22). There was a 40% increase in NBT positive neutrophils from unstimulated CREB TG and WT, which was statistically significant (p = 0.02). Neutrophil migration also was examined and there was no difference in migration between CREB TG and WT neutrophils, either untreated or treated (Figure S2). These results suggest that CREB overexpression results in increased basal NADPH oxidase activity.

## Discussion

The transcription factor CREB regulates a number of immune-related genes such as IL-2, IL-6, IL-10, TNF-alpha, cyclooxygenase-2, and macrophage migration-inhibitory factor [2], [12], [13]. Our study demonstrates that CREB overexpression in TG mice is associated with increased spontaneous skin abscess formation, decreased LPA-induced KC expression in BMDMs, and differential LPA-induced cytokine expression in BMDMs. These data suggest the possibility that CREB TG mice may have defective chemokine-mediated myeloid function and impaired innate immunity. The goal of this study was to better characterize differences between CREB TG and WT mouse abscess formation and expression of chemokines and cytokines regulating the innate immune response.

In mice, preputial glands are modified sebaceous glands that produce pheromones influencing important social features including sexual attractiveness, promotion of the estrous cycle in grouped females, dominance, and aggression [14]. Cohoused adult laboratory mice frequently display aggressive behavior [15]. Suppurative inflammation and abscesses of the preputial gland, although infrequently reported in literature, are typically long-term septic complications from fight wounds in close proximity to the external genitalia [6]. Known bacterial pathogens isolated from preputial gland abscesses include *Staphylococcus aureus, Pasteurella pneumotropica,* and *Klebsiella oxytoca* [6]–[7]. To our knowledge, this is the first description of *E. coli* preputial gland abscess formation in mice. The origin of *E. coli* in our mice is uncertain, but can likely be attributed to the close proximity of the preputial glands to the genitourinary tract and neighboring enteric flora.

Macrophages are the major source of chemokine KC that is responsible for the recruitment of neutrophils, and TLR signaling induces KC as an early essential step in the immune response to pathogen invasion [16]. Following stimulation of WT mouse BMDMs with LPA, the CXC chemokine KC and CC chemokine MCP-1 both showed increased expression. However, CREB overexpression in BMDMs had a negative effect on the induction of LPA-mediated expression and production of KC, and not MCP-1. Our data suggest that the diminished KC response to LPA stimulation may contribute to the increased incidence of skin abscess formation in CREB TG mice.

Previous studies link CREB activation to production of IL-6 in macrophages [17] and have shown that a pharmacologically increased intracellular cAMP levels using a cAMP analog activates CREB and induces IL-6 production by H19-7/IGF-IR cells [18]. We showed that LPA-stimulated BMDMs from CREB TG mice have reduced IL-1β mRNA expression and no change in IL-6 mRNA expression. It is unclear whether or not CREB overexpression affects IL-6 production early in the immune response, and whether an observed effect could be attributed directly to CREB overexpression or to diminished IL-1β-associated IL-6 induction. Further studies are needed to better characterize the differences in macrophage production of IL-1β and IL-6 at early and late phases of the immune response.

PKA mediates phosphorylation of CREB through the canonical cAMP pathway [19], and previous studies have shown that CREB binds to both TNF-α [20] and IL-10 [21] CRE promoter sites. When CRE sites in the human IL-10 promoter are mutated, transcriptional activity is lost [21] indicating that in LPS-stimulated macrophages, cAMP-activated CREB positively regulates IL-10 transcription. However, the p38-MSK1 pathway only, and not the PKA pathway, has been shown to mediate induction of both TNF-α and IL-10 in LPS-stimulated macrophages [22]. Our data did not reveal an effect of CREB overexpression on the p38-MSK1-mediated LPA-induction of TNF-α in BMDMs. Further studies are needed to evaluate whether the modest increase observed in LPA-induced IL-10 expression observed in CREB transgenic mouse BMDMs may be attributed to the p38-MSK1 pathway.

Previous literature has reported that NFκB expression may be inhibited by activated CREB through competition for rate limiting amounts of CBP/p300 [23], [24]. The balance between CREB and CBP/p300 potentially determines whether the overall response leads to respective inhibition through CREB or enhancement through CBP/p300 of NFκB activity and signaling. However, our experiments showed that expression of NFkB and a subset of its target genes were increased in unstimulated macrophages from CREB TG mice compared to WT mice. This demonstrates that CREB overexpression increases NFκB expression and activity. Furthermore, our data suggest that regulation of NFκB function appears to be independent of other chemokines induced by CREB.

To determine the effect of CREB overexpression on neutrophil function, we performed both NBT and migration assays in response to fMLP. Our results demonstrated that CREB increases basal NADPH oxidase activity but not migration. This suggests that CREB overexpressing neutrophils may be primed or are hypersensitive to stimuli such as bacteria, resulting in increased abscess formation. Others have demonstrated that altered reactive oxygen species affects the phagosome and both innate and adaptive immunity [25], [26], [27]. Further studies are necessary to determine the impact of CREB-induced increased NADPH oxidase activity and reactive oxygen species on neutrophil function and innate immunity.

In summary, this is the first report that CREB overexpression in BMDMs is associated with increased incidence of skin abscess formation. We also show that skin lesions from affected mice contain histologic evidence of acute and chronic inflammation. Detailed analysis of chemokine and cytokine expression in CREB transgenic mice reveals that CREB overexpression diminishes macrophage KC and IL-1β expression and mildly increases anti-inflammatory cytokines IL-1RA and IL-10 in a manner independent of changes to TNF-α expression. CREB also increases NFκB expression and activity. Overexpression of CREB affects basal NADPH oxidase activity in neutrophils without affecting migration. The results of this study provide new insights into the undefined role that CREB plays in immune function. Future studies will focus on characterizing macrophage infiltration and skin wound healing properties of CREB transgenic mice.

### Conclusion

Our results demonstrate that CREB overexpression in mice is associated with increased risk of preputial gland abscess formation, diminished KC production and expression, abnormal chemokine expression, and deregulated NADPH oxidase activity in neutrophils. The observed changes in chemokine and cytokine expression levels implicate dysfunctional immune signaling as potential mechanisms for the observed increase in abscess development, which require further investigation.

## Supporting Information

Figure S1 Histologic characterization of inflammatory changes in preputial gland abscesses. (A) Images from a representative CREB TG or WT mouse. The right panel shows H&E staining of a normal preputial gland from a WT mouse. The left panel shows preputial gland tissue from a CREB TG mouse with abscess formation, hyperkeratosis, and hyperplasia of the squamous epithelium (see arrows, 100X, 200X, and 400X). Magnification (400X) shows suppuration of the preputial gland, neutrophils in various stages of transmigration, and sparse infiltration of lymphocytes and plasma cells indicative of acute on chronic inflammatory conditions. Bar = 500 µm for 100x original magnification, 250 µm for 200× original magnification, and 125 µm for 400× original magnification. (B) Representative mouse with abscess of the preputial gland. Preputial gland, 500× magnification.
(PDF)

Figure S2 Neutrophil migration assay. Neutrophils were isolated from CREB TG or WT mouse bone marrow using a Percoll (Sigma, St. Louis, MO.) gradient. Cells (5–10×10^4^) were plated in the top chamber of a transwell. The bottom chamber contained media with or without 100 nM formyl-Met-Leu-Phe (fMLP). The cells were incubated at 37°C and 10% C02 for 3 hours. After incubation, the wells were washed with PBS and fixed with methanol. Wrights-Giemsa stain was performed. Each condition was performed in duplicate and counted. A representative experiment of two independent experiments is shown. WT = wild type mice, CREB = CREB transgenic mice. Error bars represent standard error.
(TIF)
