# Supplementary material for: Increased Abscess Formation and Defective Chemokine Regulation in CREB Transgenic Mice
Source: PLoS One. 2013 Feb 6;8(2):e55866. doi: 10.1371/journal.pone.0055866 (PMC3566130; doi:10.1371/journal.pone.0055866)
Supplement: Figure S1 — Histologic characterization of inflammatory changes in preputial gland abscesses. (A) Images from a representative CREB TG or WT mouse. The right panel shows H&E staining of a normal preputial gland from a WT mouse. The left panel shows preputial gland tissue from a CREB TG mouse with abscess formation, hyperkeratosis, and hyperplasia of the squamous epithelium (see arrows, 100X, 200X, and 400X). Magnification (400X) shows suppuration of the preputial gland, neutrophils in various stages of transmigration, and sparse infiltration of lymphocytes and plasma cells indicative of acute on chronic inflammatory conditions. Bar = 500 µm for 100x original magnification, 250 µm for 200× original magnification, and 125 µm for 400× original magnification. (B) Representative mouse with abscess of the preputial gland. Preputial gland, 500× magnification. (PDF) [file pone.0055866.s001.pdf]

Figure S1A

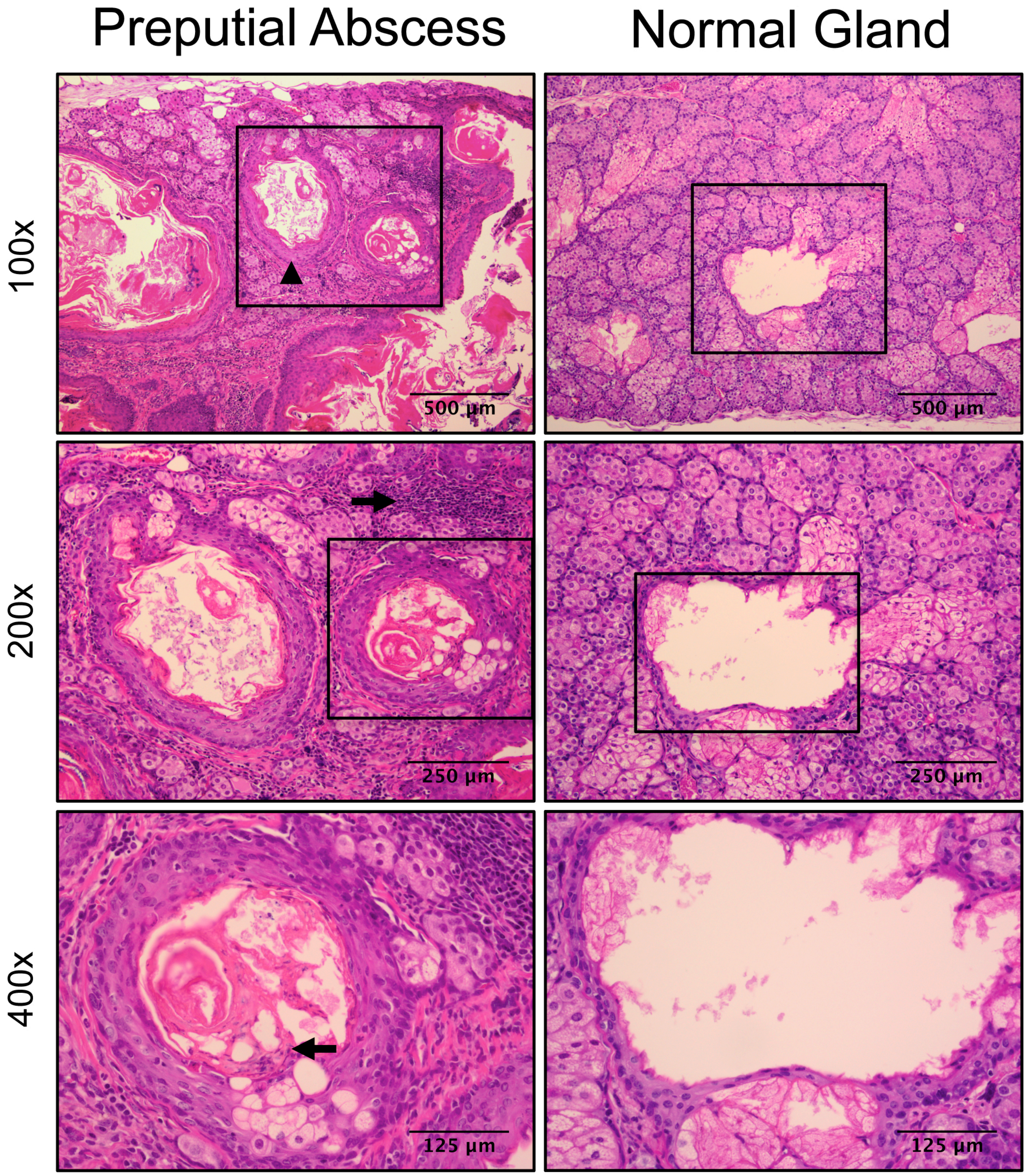
Preputial Abscess
Normal Gland
100x
200x
400x
500 μm
500 μm
250 μm
250 μm
125 μm
125 μm

Figure S1B

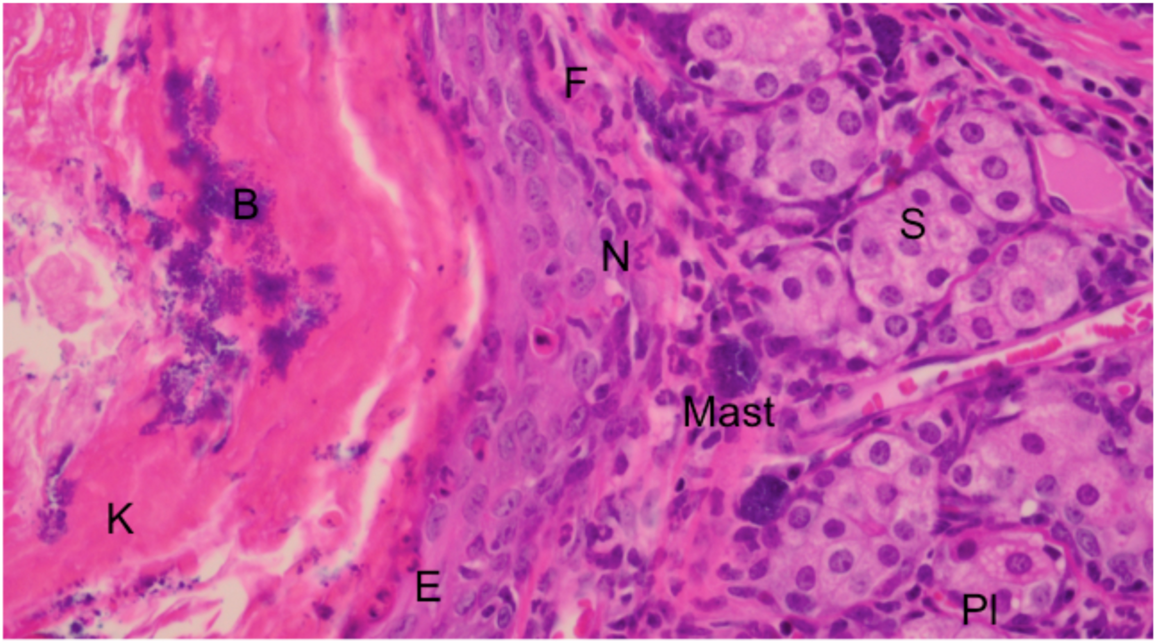

Figure Legend. Representative mouse with abscess of the preputial gland. Preputial Abscess, 500X magnification. Key:
B=Bacteria
K=Keratin production inspissated within the ducts
E=Hyperplastic duct squamous epithelium
F=Fibrosis, concentric (chronic inflammatory changed)
N=Transmigrating and migrated neutrophils
S=Sebaceous glands
Pl=Plasma cells
