# Supplementary figures and images for: Increased Abscess Formation and Defective Chemokine Regulation in CREB Transgenic Mice
Source: PLoS One. 2013 Feb 6;8(2):e55866. doi: 10.1371/journal.pone.0055866 (PMC3566130; doi:10.1371/journal.pone.0055866)

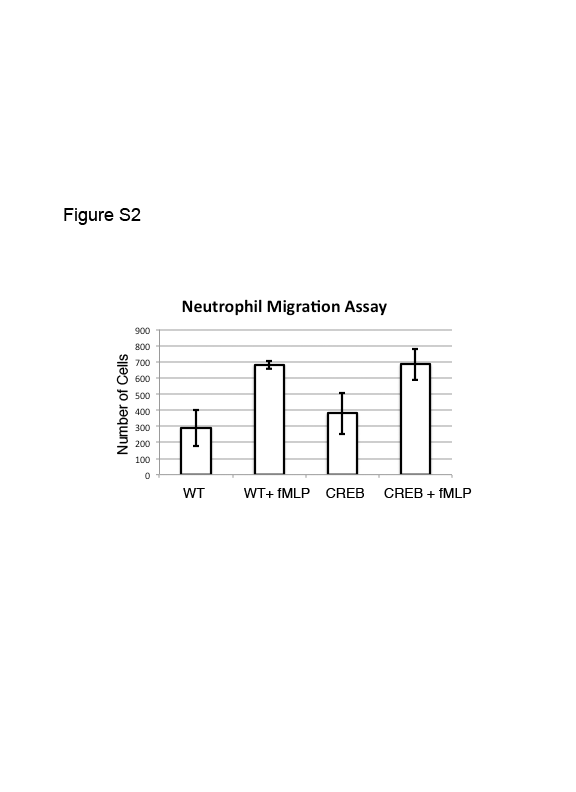

Supplement: Figure S2 — Neutrophil migration assay. Neutrophils were isolated from CREB TG or WT mouse bone marrow using a Percoll (Sigma, St. Louis, MO.) gradient. Cells (5–10×104) were plated in the top chamber of a transwell. The bottom chamber contained media with or without 100 nM formyl-Met-Leu-Phe (fMLP). The cells were incubated at 37°C and 10% C02 for 3 hours. After incubation, the wells were washed with PBS and fixed with methanol. Wrights-Giemsa stain was performed. Each condition was performed in duplicate and counted. A representative experiment of two independent experiments is shown. WT = wild type mice, CREB = CREB transgenic mice. Error bars represent standard error. (TIF) [file pone.0055866.s002.tif]
